# A possible link between olfaction and miscarriage

**DOI:** 10.7554/eLife.62534

**Published:** 2020-09-29

**Authors:** Neven Borak, Johannes Kohl

**Affiliations:** State-dependent Neural Processing Lab, Francis Crick InstituteLondonUnited Kingdom

**Keywords:** Bruce effect, pregnancy block, miscarriage, repeated pregnancy loss, olfaction, social chemosignaling, Human

## Abstract

Unexplained repeated pregnancy loss is associated with an altered perception of male odors and differences in brain regions that process smells.

**Related research article** Rozenkrantz L, Weissgross R, Weiss T, Ravreby I, Frumin I, Shushan S, Gorodisky L, Reshef N, Holzman Y, Pinchover L, Endevelt-Shapira Y, Mishor E, Soroka T, Finkel M, Tagania L, Ravia A, Perl O, Furman-Haran E, Carp H, Sobel N. 2020. Unexplained repeated pregnancy loss is associated with altered perceptual and brain responses to men’s body-odor. *eLife* **9**:e55305. doi: 10.7554/eLife.55305

Unexplained repeated pregnancy loss is a poorly understood condition that can cause significant distress and for which no effective treatment exists. Much research to date has focused on dysfunctions of the uterus or hormonal signaling (Saravelos and Regan, 2014), but the possible involvement of the nervous system has not been explored despite the role of the olfactory system in mammalian reproduction being well-documented (Dulac and Torello, 2003).

Exposing female rodents to the smell of adult males can lead to synchronized menstrual cycling (Whitten, 1956) and accelerated sexual maturation (Vandenbergh, 1967), as well as to embryos failing to implant in the uterus (Bruce, 1959). Olfactory cues might also play a role in human reproduction: for instance, the menstrual cycle phase may influence preferences for male odors (Rantala et al., 2006). This presents the possibility that altered neural processing of socially relevant odors, such as the scent of a partner, may be linked to pregnancy loss and other reproductive disorders. Now, in eLife, Noam Sobel (Weizmann Institute of Science) and colleagues – including Liron Rozenkrantz, Reut Weissgross and Tali Weiss as joint first authors – report that women who have experienced unexplained repeated pregnancy loss process the odors of males differently (Rozenkrantz et al., 2020).

The researchers started by measuring the ability of women to discern the body odor of their spouse when presented with three choices – a blank odor, their spouse's odor and a non-spouse odor. Women who had experienced unexplained repeated pregnancy loss (the uRPL group) were almost twice as likely to be able to recognize their spouse’s odor as women who had not experienced the condition (the control group). Indeed, women in the control group performed no better than would be expected by chance (Figure 1A). Might this be due to women in the uRPL group simply having a better sense of smell? Rozenkrantz et al. found that women in the uRPL group were only marginally better at discerning odors in general, which suggests that the observed effect may be specific to smells that are socially important. Moreover, women in the uRPL group rated non-spousal odors differently from women in the control group in terms of perceived intensity, pleasantness, sexual attraction and fertility, again indicating that olfactory processing is altered in women who have experienced the condition.

**Figure 1.**
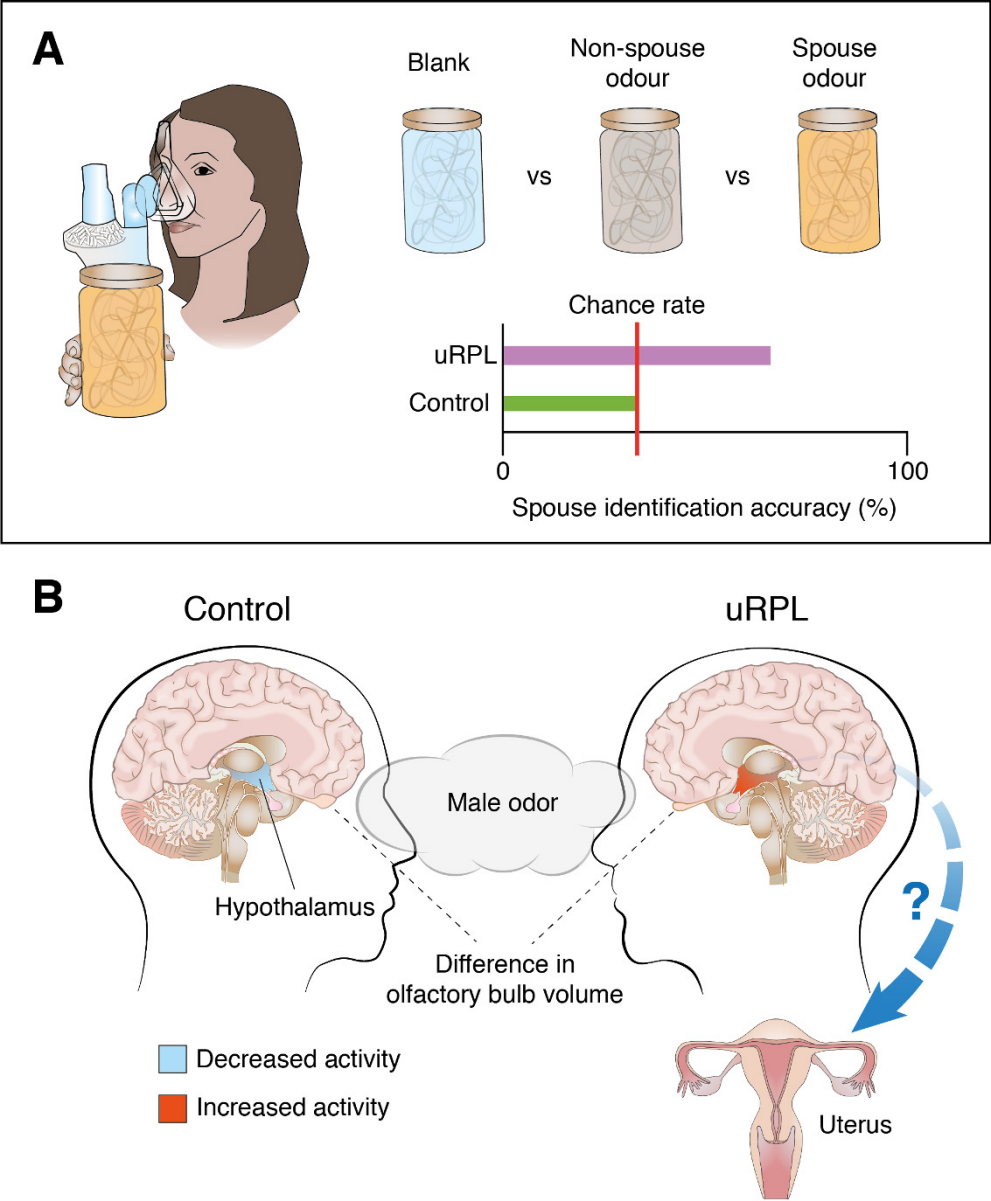
Differences in how women who have experienced unexplained repeated pregnancy loss (uRPL) and women without the condition perceive odors, and how their brains process them. (**A**) A group of women who have experienced uRPL and a control group were exposed to three different odors: a blank odor; the odor of their spouse; and a non-spouse odor. The number of women in the control group able to identify their partner’s odor could be explained by chance; the number of women in the uRPL group able to identify their partner’s odor was significantly higher. (**B**) Exposure to subliminal levels of male body odor while watching arousing movie clips elicits increased activity in the hypothalamus of women in the uRPL group (indicated in red; right), whereas hypothalamic activity decreases slightly in the control group (pale blue; left). Women in the uRPL group also had smaller olfactory bulbs. However, the precise links between altered olfactory processing in the brain and miscarriage are not yet fully understood.

Image credit: Joe Brock, Francis Crick Institute.

Rozenkrantz et al. next asked whether these differences in olfactory performance and perception were also reflected in brain form and function. Magnetic resonance imaging (MRI) showed that the olfactory bulb – the first brain structure that relays olfactory information from the nose – was significantly smaller in women in the uRPL group than in women in the control group (Figure 1B). This is a striking finding since smaller olfactory bulbs have previously been linked to poorer olfactory performance (Rombaux et al., 2010).

Rozenkrantz et al. next addressed whether the observed differences in the perception of male odors were reflected in altered brain activity. For this purpose, women from the uRPL and control groups were presented with non-spouse male odors while watching arousing movie clips in an MRI scanner. The initial analysis focused on the hypothalamus, a small but critical structure deep in the brain that links olfactory and reproductive functions. Rozenkrantz et al. found that activity in the hypothalamus increased in women from the uRPL group exposed to non-spouse male odor, while it decreased in women from the control group (Figure 1B). Additionally, exposure to non-spouse male odor increased correlated activity between the hypothalamus and the insula (an area in the cortex) significantly more in the control group than in the uRPL group. The link between these two brain areas may facilitate odor-based kin recognition (Lundström et al., 2009).

Overall, these experiments provide compelling evidence that women who have experienced unexplained repeated pregnancy loss perceive socially important odors in a different way, and that this is associated with specific changes in brain structure and function. However, this latest work does not reveal causal links between olfaction and the condition, and further research is needed to establish whether these differences in perception arise as a consequence of experiencing multiple miscarriages or whether they are present beforehand.

Rozenkrantz and colleagues – who are based at the Weizmann Institute of Science, the Edith Wolfson Medical Center and the Sheba Medical Center – also discuss conceptual similarities to the Bruce effect, a phenomenon first described in rodents, where pregnancy is terminated in females exposed to the scent of an unfamiliar male (Bruce, 1959). Since women in the uRPL group perceived non-spouse odors differently, this could be linked to an increased risk of miscarriage via a similar mechanism. However, the Bruce effect in rodents relies on brain structures that are nonexistent or vestigial in humans, casting doubts on the existence of this phenomenon in humans. Finally, it is also possible that partners of women experiencing unexplained repeated pregnancy loss might have a unique scent that contributes to repeated miscarriages. Exploring the possible mechanisms linking olfaction with unexplained pregnancy loss will thus be an important direction for future studies.
